# Music therapy and music-based support for autistic children: a neurodiversity-informed systematic review and meta-analysis

**DOI:** 10.3389/fpsyt.2026.1806096

**Published:** 2026-10-09

**Authors:** Shanshan Lu, Huimin Feng, Zhidan Wang

**Affiliations:** 1 Xuzhou Kindergarten Teachers College, Xuzhou, China; 2 School of Education Science, Jiangsu Normal University, Xuzhou, China

**Keywords:** autism, meta-analysis, moderator, music therapy, music-based intervention, neurodiversity

## Abstract

**Background:**

Music-based interventions, including music therapy, are widely used to support autistic children, but reductions on autism-related scales do not fully represent communication, participation, quality of life, or autonomy. We retained clinically interpretable outcomes while using a neurodiversity-informed framework to organize intervention goals and interpretation.

**Methods:**

Web of Science, CNKI, Wanfang Data, VIP, PubMed, the Cochrane Library, Embase, Wiley Online Library, EBSCOhost, and Google Scholar were searched from inception to August 5, 2026. Randomized, quasi-randomized, crossover, and controlled studies involving autistic participants younger than 18 years were eligible. Outcomes were grouped as social communication/interaction, language, adaptive function/participation, quality of life/well-being, and autism-related characteristics and support needs. Positive Hedges’ g values favored the music-based condition. Random-effects models used restricted maximum likelihood estimation and modified Hartung-Knapp inference. Comparator intensity, technique, duration, and parent involvement were examined as exploratory moderators.

**Results:**

Thirty-three independent studies, representing 1,880 participants in the selected quantitative comparisons, were included and contributed at least one outcome. Social communication/interaction showed a significant pooled effect (k = 10, g = 0.64, 95% CI 0.25 to 1.03, p = 0.0046; I² = 59.6%); the primary evidence layer also remained significant (k = 7, g = 0.57, 95% CI 0.02 to 1.12, p = 0.043). Language showed a significant small pooled effect (k = 11, g = 0.32, 95% CI 0.10 to 0.54, p = 0.009; I² = 0%), whereas the primary evidence layer yielded g = 0.19 (95% CI -0.17 to 0.55). Autism-related scale outcomes showed a significant pooled effect (k = 16, g = 1.23, 95% CI 0.93 to 1.53, p < 0.001; I² = 77.0%) and remained significant among studies reporting a reproducible randomization method (k = 6, g = 0.95, 95% CI 0.29 to 1.61). Adaptive function and quality-of-life estimates did not reach statistical significance. No between-group moderator test was significant. Duration was not associated with effect size (β = -0.034 per month, p = 0.552), and Egger’s regression did not detect funnel-plot asymmetry (p = 0.591).

**Conclusions:**

Music-based support produced favorable average effects on social communication/interaction, language, and autism-related scale outcomes.

## 1 Introduction

Autism is a lifelong neurodevelopmental difference associated with wide variation in communication, sensory processing, learning, daily living, health, and support needs. Over the past three decades, autism research has increasingly engaged with the neurodiversity movement, which was shaped largely by autistic self-advocates. Within this framework, autism is understood not only through impairment but also as a form of human neurological variation; research and services should respect autistic identity, agency, and priorities (1–3). This position does not minimize disability, distress, intellectual disability, co-occurring health conditions, or the need for effective support. It shifts the central question from normalization to reducing barriers, responding to distress, expanding communication and participation, and improving quality of life for children and families.

The relationship between neurodiversity and intervention remains contested. Neurodiversity-informed scholars have noted that intervention goals can encode neurotypical expectations, particularly when success is defined by suppressing visible autistic behavior. A clinical-developmental perspective emphasizes that children and families may reasonably seek help with communication, self-injury, anxiety, sleep, safety, learning, adaptive functioning, and participation, and that such support need not erase autistic identity (4). These positions are compatible when goals are chosen carefully: an intervention may reduce distress or remove a barrier while respecting the child, and affirming language does not by itself demonstrate effectiveness. A systematic review should therefore report what was measured, examine whether each outcome fits the proposed mechanism and goal, and give equal attention to beneficial, null, and adverse findings.

Music is especially relevant to this discussion. Musical interest and ability are not universal among autistic people, but some samples have shown relative strengths in pitch discrimination, melodic memory, and the processing of musical structure (5–8). Music can provide predictable timing, repetition without exact sameness, multimodal sensory input, nonverbal expression, and opportunities for shared attention and turn-taking. For some children, these features may make musical interaction accessible and rewarding when spoken interaction is effortful. They also suggest plausible mechanisms involving arousal regulation, temporal prediction, auditory-motor coupling, social contingency, and reward (9–12).

The literature uses the terms music therapy, music-based intervention, music-assisted training, and related labels inconsistently. We use *music-based support* as an inclusive term and retain music therapy when it reflects the terminology and professional context of the primary study. Eligible interventions used musical activity as a constitutive component of a structured therapeutic, developmental, or participation-oriented process, including improvisation, singing, instrument play, movement, composition, or receptive listening. Auditory integration training and Tomatis-type protocols were excluded because they modify or filter sound input and rest on a different theory of action. Music education was also excluded when the primary aim was the acquisition of musical knowledge or performance skills.

A central methodological problem is the definition of benefit. Many studies use the Social Responsiveness Scale (SRS), Autism Behavior Checklist (ABC), Childhood Autism Rating Scale (CARS), Autism Treatment Evaluation Checklist (ATEC), or related instruments. These measures support standardized comparisons and may include items that are relevant to everyday support needs. However, their total scores combine heterogeneous behaviors, some items may index difference rather than harm, and a lower score cannot automatically be interpreted as greater well-being or a more valued life. The converse position is also unhelpful: rejecting every established measure would make unfavorable findings difficult to interpret. A balanced framework should retain clearly labeled autism-related measures while also assessing quality of life, participation, functional communication, adaptive function, family experience, goal attainment, and adverse events (13).

Concerns about outcome selection are well established in autism intervention research. Autistic self-advocacy and neurodiversity scholarship have called for participatory selection of outcomes that matter to autistic people and families (2, 3). Meta-research has further shown that estimated effects vary with outcome proximity, reporter, measurement context, and risk of detection bias (14). In music therapy, a previous review found no clear immediate difference in generalized social interaction or verbal and nonverbal communication, but reported more favorable evidence for global improvement, quality of life, and overall autism-related severity (15). An integrative review likewise identified poor alignment among intervention approaches, stated goals, and outcome measures (16). Goal-outcome alignment is therefore a methodological requirement, not a reason to disregard unfavorable results.

Previous reviews have established that music-based interventions warrant continued study, but most have organized evidence around symptom scales or broad treatment labels. Less attention has been given to the alignment between intervention goals and outcomes, to the distinction between proximal engagement and generalized functioning, or to the mechanisms represented by common moderator categories. The present review addresses this gap by combining a neurodiversity-informed outcome architecture with source-verified effect extraction and theory-led moderator analyses. Its purpose is not to privilege favorable outcomes, but to make the meaning and limits of each effect estimate more explicit.

### 1.1 Factors that may influence outcomes of music-based support

Clinical and methodological heterogeneity makes a single average effect incomplete. Moderator analysis was organized around a mechanism chain rather than a set of convenient study descriptors: developmental stage and musical accessibility may influence entry into the intervention; engagement mode and therapist-child contingency may shape proximal interaction; duration and parent participation may affect consolidation and transfer; and comparator intensity determines what the estimated effect represents. Candidate variables were coded without reference to effect size and evaluated with between-group tests rather than the significance of individual subgroups. Because the review was not prospectively registered and study-level information was limited, all moderator analyses were treated as exploratory.

#### 1.1.1 Developmental stage and age

Developmental stage may shape both the goals and mechanisms of music-based support. In early childhood, musical routines may scaffold shared attention, imitation, preverbal turn-taking, and parent-child synchrony; during the school years, goals may extend to peer participation, self-expression, language learning, emotion regulation, and educational engagement. Meta-analyses of early autism intervention suggest that developmental timing and baseline ability may be associated with communication outcomes, but do not support a universal age threshold (14, 17). Because study-level mean age can obscure substantial within-study variation, age was summarized descriptively rather than dichotomized.

#### 1.1.2 Modality and engagement mechanism

Music-based support may involve active, receptive, or mixed activities. Active approaches use singing, instruments, movement, improvisation, or composition to engage agency, social contingency, and auditory-motor coupling. Receptive approaches may use predictable musical structure to support attention or regulation, whereas mixed approaches combine listening, active response, and relational interaction (18, 19). Because most trials combined several activities, coding followed the dominant engagement mechanism rather than treating technique labels as pure, mutually exclusive treatment brands.

#### 1.1.3 Technique and therapist-child interaction

Specific approaches included free or structured improvisation, song-based language activities, Orff-based instrument and movement work, piano-centered programs, and structured listening. Outcomes may differ because these approaches vary in therapist responsiveness, musical contingency, predictability, repetition, and opportunities for child initiation (20–22). Technique labels may also proxy therapist training, setting, culture, comparator, and outcome selection. We therefore required a statistically reliable between-group test before interpreting technique differences and treated such findings as hypothesis-generating rather than evidence of a superior treatment.

#### 1.1.4 Intervention duration and dose

Duration could plausibly influence outcomes in opposing ways. Repetition may be needed to establish a therapeutic relationship, learn musical routines, and support transfer, whereas novelty may strengthen early engagement and attendance may decline in longer programs. Calendar duration is not equivalent to cumulative dose because session length, frequency, attendance, and fidelity vary. Duration in months was therefore modeled continuously. A three-month split was retained only as a descriptive summary because approximately 12 weeks was common among shorter programs; it was not used to identify an optimal duration.

#### 1.1.5 Parent involvement, delivery context, and comparator intensity

Parent involvement may promote generalization by embedding musical routines in family interaction and helping caregivers recognize the child’s communicative signals (22, 23). Individual and group delivery may also differ in relational demands and opportunities for peer participation. These factors were explored only when coding was sufficiently clear. Comparator intensity was examined as a methodological moderator because usual care, no additional intervention, matched nonmusic activity, and intensive communication programs control for different combinations of therapist attention, expectancy, structure, and social opportunity. Risk of bias was examined in sensitivity analyses rather than treated as a clinical moderator, because it was confounded with outcome scale, comparator, country, and reporting practices.

### 1.2 Objectives

This review makes two linked contributions. First, it applies a neurodiversity-informed outcome framework without discarding clinically interpretable autism-related measures: scale change is analyzed separately from communication, participation, adaptive function, and quality of life. Second, it connects exploratory moderators to hypothesized mechanisms of access, interaction, consolidation, transfer, and causal contrast rather than treating subgroup labels as self-explanatory. The specific aims were to (1) estimate domain-specific effects; (2) compare usual or enhanced care with active comparators; (3) examine technique, duration, and parent participation; and (4) assess robustness through risk-of-bias restrictions, randomization-reporting restrictions, and leave-one-out analyses. The framework guided terminology, outcome organization, and interpretation; it did not alter eligibility criteria or predetermine the direction of the findings.

## 2 Methods

### 2.1 Information sources and search strategy

This systematic review and meta-analysis was conducted in accordance with the Preferred Reporting Items for Systematic Reviews and Meta-Analyses (PRISMA) guidelines (24). Web of Science, CNKI (China National Knowledge Infrastructure), Wanfang Data, VIP (China VIP Information), PubMed, the Cochrane Library, Embase, Wiley Online Library, EBSCOhost, and Google Scholar were searched from inception to August 5, 2026.

English-language searches combined controlled vocabulary and free-text terms for autism (including autis*, Asperger*, and pervasive developmental disorder) with terms for music therapy and music-based intervention (including music therap*, music-based, musical intervention, improvisational music, active music, and receptive music). Chinese-language searches combined terms for autism with music therapy, music intervention, Orff, and improvisational music.

### 2.2 Eligibility criteria

Studies were eligible if they (1) included autistic participants younger than 18 years, with child data separately extractable from mixed-age samples; (2) used a randomized, quasi-randomized, crossover, or controlled comparative design; (3) evaluated a structured music-based intervention in which musical activity was a constitutive component of the experimental contrast; (4) included usual care, enhanced care, no additional intervention, an active nonmusic activity, or another analyzable comparator; and (5) reported data sufficient to calculate or recover an effect estimate. Eligible outcomes included quality of life or well-being, participation, social communication or interaction, language, adaptive function, family or caregiver outcomes, adverse events, and clearly labeled measures of autism-related characteristics or support needs.

Studies were excluded from the quantitative synthesis if the sample was adult-only; the report duplicated or overlapped an earlier sample; the design was uncontrolled, a case series, or a single-case design outside the review definition; the intervention was auditory integration training or a Tomatis-type modified-sound protocol; the music component could not be isolated from a multicomponent contrast; both groups received music and the contrast therefore concerned music format rather than music-specific effectiveness; or the variance required for analysis could not be recovered.

Mixed child-adult samples were eligible only when child data were reported separately. Multiple reports from the same cohort were linked and counted as one study, and the earliest, most complete source was used for quantitative extraction.

### 2.3 Study selection, data extraction, and source verification

Two researchers independently assessed titles and abstracts and then full texts against the eligibility criteria. A structured form captured study identity, year, country or setting, design, analyzed sample sizes, participant age, intervention content, comparator, session schedule, parent involvement, delivery format, outcome instrument, reporter, time point, scoring direction, means, standard deviations, change scores, author-reported standardized effects, and risk-of-bias information. Reports from the same cohort were linked before quantitative synthesis.

Data were extracted independently by the same two researchers. Disagreements in study selection or extraction were resolved through discussion and, when necessary, consultation with a third researcher. Change scores were preferred when clearly reported; otherwise, endpoint data or author-reported standardized effects were used. Standard deviations were recovered from confidence intervals only when the interval definition and sample size were explicit. Crossover trials were analyzed at the participant rather than session level, and analyzed sample sizes after attrition were used.

When several eligible outcomes were available, effects were first separated by domain, reporter, comparator, and time point. Within each synthesis, one effect was selected per independent study sample to avoid double counting. In-session interaction measures were not pooled with generalized parent- or clinician-rated outcomes.

### 2.4 Outcome domains and direction of effect

The primary domains were (1) social communication/interaction, (2) language, (3) adaptive function/participation, and (4) quality of life/well-being. Autism-related scales were retained in a separate domain, labeled autism-related characteristics and support needs, and were not relabeled as well-being or personal development. When lower scores indicated fewer measured difficulties, the sign was reversed so that a positive Hedges’ g consistently favored the music-based condition. Risk of bias, comparator intensity, and the reporting of randomization methods were examined only in subgroup or sensitivity analyses.

### 2.5 Coding of intervention and moderator variables

The dominant engagement mechanism was coded as active, mixed/interactive, or receptive/visual from the intervention description, without reference to the observed effect size. Duration was coded in months; continuous meta-regression was the primary duration analysis, whereas the ≤3-month versus >3-month split was descriptive. Parent involvement required a core parent-child component. Comparator coding distinguished usual or enhanced care, no additional intervention, active non-music intervention, and active music or developmental comparison. Mean age was incompletely reported; consequently, no age meta-regression was performed. All moderator categories were derived from intervention and design information rather than from the pattern of study results.

### 2.6 Risk of bias

Randomized studies were appraised at the outcome level using RoB 2, and nonrandomized studies were appraised at the outcome level using ROBINS-I, covering randomization or allocation, deviations from intended intervention, missing outcome data, outcome measurement, and selective reporting (25). Two researchers assessed risk of bias independently; disagreements were resolved by consensus or consultation with a third researcher. Missing methodological information was not treated as evidence of adequate practice. Risk of bias informed sensitivity analyses and was neither converted to a summed quality score nor inferred from country or publication language.

### 2.7 Effect-size calculation and synthesis

Continuous outcomes were converted to Hedges’ g and its sampling variance (26). For parallel-group studies, standardized mean differences were calculated from group means and pooled standard deviations and then multiplied by the small-sample correction. Author-reported standardized effects were converted to Hedges’ g using the corresponding group sizes. Standard deviations reconstructed from confidence intervals were used only when the report defined the interval and sample size clearly. Separate meta-analyses were conducted for each outcome domain.

Random-effects models were specified *a priori* because clinical and methodological heterogeneity was expected. Between-study variance was estimated by restricted maximum likelihood. Confidence intervals used a modified Hartung-Knapp approach, which can produce substantially wider intervals than normal-approximation methods when few studies are available. Cochran’s Q, τ², and I² were reported, but I² was not used mechanically to select the model. A 95% prediction interval was reported when at least three studies were available.

### 2.8 Moderator, publication-bias, and sensitivity analyses

Categorical moderators were evaluated with between-group Q tests under a common residual heterogeneity estimate. Moderation was judged from Qbetween and its p value, not from whether an individual subgroup reached statistical significance. Duration was examined by random-effects meta-regression in months. Analyses of technique, parent involvement, duration, and comparator were exploratory.

Funnel plots and Egger’s regression test were used only for syntheses containing at least 10 independent studies (27). Influence was examined with leave-one-out analyses. The social-communication synthesis was repeated after excluding studies at high risk of bias or with major methodological concerns; autism-related scale outcomes were repeated among studies that reported a reproducible randomization method.

## 3 Results

### 3.1 Study selection and audit

The study selection process is summarized in **Figure 1**. The review included 33 independent studies, all of which contributed at least one quantitative effect. Counting each crossover participant once and using the participants entering each study’s selected quantitative contrast, 1,880 participants contributed at least one outcome. Adult samples, overlapping reports, auditory integration or Tomatis interventions, and nonisolable contrasts were excluded from the music-specific synthesis.

**Figure 1:**
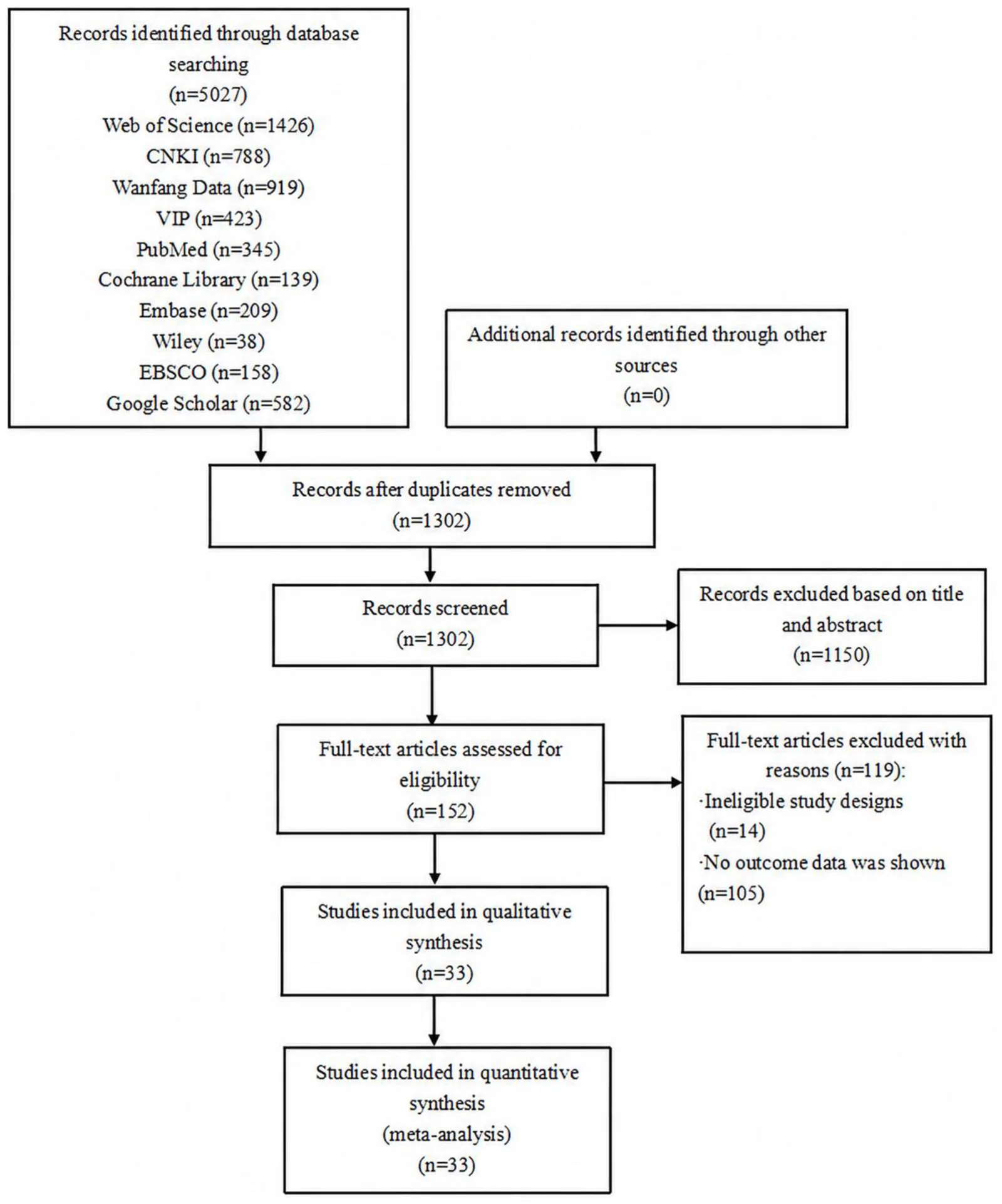
Flowchart of the literature selection process.

### 3.2 Study characteristics and coding

The characteristics of all 33 studies included in the quantitative synthesis are summarized in **Table 1**. The evidence ranged from small crossover and feasibility studies to multicenter randomized trials. Music-based conditions included improvisational, family-centered, and parent-child music therapy; relational and group social-skills music therapy; singing paired with gestures; Orff instruments and movement; piano activities; structured listening; and music-assisted language learning (30, 35, 38, 41, 42). Comparators included usual or enhanced care, no additional intervention, nonmusic play or verbal activities, and active developmental or communication programs.

**Table 1:** Characteristics of all 33 studies included in the quantitative synthesis.

Study	Design; autistic participants, music/comparator (n)	Music condition	Comparator	Outcome domain(s)
Arezina (28)	Randomized crossover; n=6 (same participants)	Relational/improvisational	Within-person nonmusic interactive play	Social communication/interaction
Bieleninik et al. (29)	Assessor-blinded multicentre RCT; n=142/129	Relational/improvisational	Enhanced standard care	Social communication/interaction
Buday (30)	Randomized-order crossover; n=10 (same participants)	Song/language-targeted	Active rhythmic-speech condition	Language
Chen et al. (31)	Controlled; randomization reported/unclear; n=15/15	Comprehensive music activities	Same-background usual care	Autism-related characteristics/support needs
Chen et al. (32)	Controlled; randomization reported/unclear; n=46/50	Visual/receptive music	Same-background usual care	Autism-related characteristics/support needs
Dănciulescu & Zaharia (33)	Availability-allocated quasi-trial; n=30/30	Instrumental/piano	Standard care (ABA + speech)	Language
Farmer (34)	Very small RCT; n=5/5	Song/language-targeted	Identical nonmusic gestures	Language
Gattino et al. (35)	Assessor-blinded RCT; n=12/12	Relational/improvisational	Usual care	Social communication/interaction; Language
Ghasemtabar et al. (36)	Matched clinical trial; n=13/14	Orff/group music	No additional intervention	Social communication/interaction
He et al. (23)	Controlled; randomization reported/unclear; n=50/50	Parent-child comprehensive music	Same-background usual care	Autism-related characteristics/support needs
Huang (37)	Controlled; randomization reported/unclear; n=7/5	Instrumental/Orff	Same-background usual care	Autism-related characteristics/support needs
LaGasse (38)	Randomized active-control trial; n=8/8	Structured group social-skills music	Active nonmusic social-skills group	Social communication/interaction
Li et al. (39)	Controlled; randomization reported/unclear; n=40/40	Music plus language training	Same-background usual care	Autism-related characteristics/support needs
Li et al. (40)	Controlled; randomization reported/unclear; n=47/47	Active music activities	Same-background usual care	Autism-related characteristics/support needs
Lim (41)	Randomized three-arm parallel trial; n=18/18; third arm n=14	Song/language-targeted	Active speech-language training	Language
Lim and Draper (42)	Randomized-order within-participant trial; n=22 (same participants)	Song/language-targeted	Active speech ABA-VB training	Language
Liu et al. (43)	Single-blind RCT; n=19/19	Song/language-targeted	Speech therapy	Language
Nie et al. (44)	Controlled; randomization reported/unclear; n=35/35	Orff plus floor-time	Same-background usual care	Autism-related characteristics/support needs
Rabeyron et al. (45)	RCT; n=19/17	Structured plus improvisational music	Music listening	Autism-related characteristics/support needs
Sharda et al. (12)	Assessor-blinded RCT; n=24-26/23-25	Individual active music	Matched nonmusic intervention	Social communication/interaction; Quality of life/wellbeing; Language; Adaptive function/participation
Shen et al. (46)	Controlled; randomization reported/unclear; n=38/37	Parent-child comprehensive music	Same-background usual care	Autism-related characteristics/support needs
Sui (47)	Controlled; randomization reported/unclear; n=35/35	Active music activities	Same-background usual care	Autism-related characteristics/support needs
Thompson et al. (22)	RCT; n=11/10	Family-centred/relational	Usual care	Social communication/interaction; Language
Wan (48)	Controlled; randomization reported/unclear; n=40/40	Individualised active or receptive music	Same-background usual care	Autism-related characteristics/support needs
Wang (49)	Comparative study; n=40/40	Songs, instruments and improvisation	FloorTime active comparator	Autism-related characteristics/support needs
Wang et al. (50)	Controlled; randomization reported/unclear; n=26/26	Comprehensive music activities	Same-background usual care	Autism-related characteristics/support needs
Wen (51)	Nonrandom historical control; n=37/37	Song-based social stories	Historical rehabilitation control	Quality of life/wellbeing; Autism-related characteristics/support needs
Williams et al. (52)	Feasibility RCT; n=11/8	Parent-mediated song/language	Intensive nonmusic communication intervention	Social communication/interaction; Language; Adaptive function/participation
Yum et al. (53)	Mixed individual/cluster RCT; n=33/34	Interactive group music	Active nonmusic social-skills intervention	Social communication/interaction
Yurteri et al. (54)	Small controlled trial; n=12/12	Relational/improvisational	Usual care	Quality of life/wellbeing
Zhou (55)	Controlled; randomization reported/unclear; n=48/48	Song/language-targeted	Same-background usual care	Autism-related characteristics/support needs
Zhou et al. (56)	Controlled; randomization reported/unclear; n=53/55	Comprehensive music activities	Same-background usual care	Autism-related characteristics/support needs
Zhou et al. (57)	RCT; n=15/14	Song/language-targeted	Usual care	Social communication/interaction; Language; Adaptive function/participation

Risk of bias was assessed using RoB 2 for randomized studies and ROBINS-I for nonrandomized studies. Among the 28 studies appraised with RoB 2, one was classified as low risk, 23 as having some concerns, and four as high risk for the selected outcomes. The five nonrandomized studies appraised with ROBINS-I were classified as serious risk. Recurring concerns included incomplete reporting of sequence generation and allocation concealment, unblinded parent or therapist ratings, unclear handling of missing data, limited reporting of co-interventions, and possible selective reporting. Risk of bias was not assigned on the basis of country or publication language.

### 3.3 Outcome-specific meta-analyses

Social communication/interaction. Across 10 studies, the pooled effect was g = 0.64 (95% CI 0.25 to 1.03, p = 0.0046; τ² = 0.154; I² = 59.6%), with a 95% prediction interval of -0.35 to 1.63. The primary evidence layer yielded g = 0.57 (95% CI 0.02 to 1.12, p = 0.043; k = 7). Domain-level pooled estimates are summarized in **Table 2**.

**Table 2:** Random-effects meta-analyses by outcome domain.

Outcome domain	k	g	95% CI	p	tau²	I² (%)	95% prediction interval
Social communication/interaction	10	0.64	0.25 to 1.03	0.005	0.15	59.6	-0.35 to 1.63
Language	11	0.32	0.10 to 0.54	0.009	0.00	0.0	0.09 to 0.54
Adaptive function/participation	3	0.47	-1.06 to 1.99	0.320	0.24	64.7	-7.24 to 8.17
Quality of life/wellbeing	3	0.94	-0.29 to 2.16	0.081	0.15	62.3	-5.11 to 6.99
Autism-related characteristics/support needs	16	1.23	0.93 to 1.53	<0.001	0.24	77.0	0.14 to 2.31

Positive Hedges’ g values favor the music-based condition.

Language. Eleven studies yielded a significant small effect, g = 0.32 (95% CI 0.10 to 0.54, p = 0.009; τ² = 0; I² = 0%), with a 95% prediction interval of 0.09 to 0.54. The primary evidence layer yielded g = 0.19 (95% CI -0.17 to 0.55, p = 0.242; k = 6).

Adaptive function/participation. Three studies yielded g = 0.47 (95% CI −1.06 to 1.99, p = 0.320; I² = 64.7%).

Quality of life/well-being. Three studies yielded g = 0.94 (95% CI −0.29 to 2.16, p = 0.081; I² = 62.3%).

Autism-related characteristics and support needs. Sixteen studies yielded g = 1.23 (95% CI 0.93 to 1.53, p < 0.001; τ² = 0.236; I² = 77.0%), with a 95% prediction interval of 0.14 to 2.31. Restricting the analysis to six studies that reported a reproducible randomization method produced g = 0.95 (95% CI 0.29 to 1.61, p = 0.014; I² = 83.1%).

### 3.4 Exploratory moderator analyses

For social communication/interaction, the usual or enhanced-care subgroup yielded g = 0.74 (95% CI -0.06 to 1.53; k = 5), whereas active nonmusic comparators yielded g = 0.55 (95% CI -0.05 to 1.15; k = 5). The between-group difference was not significant (Qbetween = 0.08, p = 0.774) (**Table 3**).

**Table 3:** Exploratory moderator analyses.

Moderator	Level/slope	k	g or β	95% CI	Within p	Qbetween	Between p
Social communication: comparator	Active non-music comparator	5	0.55	-0.05 to 1.15	0.064	0.08	0.774
Social communication: comparator	Usual/enhanced care	5	0.74	-0.06 to 1.53	0.063	0.08	0.774
Autism-related outcomes: technique	Active	7	1.17	0.70 to 1.64	<0.001	0.27	0.873
Autism-related outcomes: technique	Mixed/interactive	6	1.33	0.50 to 2.15	0.009	0.27	0.873
Autism-related outcomes: technique	Receptive/visual	3	1.20	0.32 to 2.09	0.028	0.27	0.873
Autism-related outcomes: comparator	Active music/developmental comparator	2	0.83	-7.99 to 9.66	0.442	0.94	0.331
Autism-related outcomes: comparator	Usual/background care	14	1.28	0.98 to 1.58	<0.001	0.94	0.331
Autism-related outcomes: parent involvement	False	13	1.30	0.97 to 1.62	<0.001	1.12	0.289
Autism-related outcomes: parent involvement	True	3	0.91	-0.80 to 2.62	0.150	1.12	0.289
Autism-related outcomes: duration descriptive	<=3 months	8	1.37	0.80 to 1.93	<0.001	0.89	0.347
Autism-related outcomes: duration descriptive	>3 months	8	1.11	0.74 to 1.48	<0.001	0.89	0.347
Autism-related outcomes: duration meta-regression	Per additional month	16	-0.03	-0.15 to 0.08	—	—	0.552

Statistical significance within a subgroup does not establish a difference between subgroups.

For autism-related scale outcomes, pooled effects were significant within the active (k = 7, g = 1.17, 95% CI 0.70 to 1.64, p < 0.001), mixed/interactive (k = 6, g = 1.33, 95% CI 0.50 to 2.15, p = 0.009), and receptive/visual (k = 3, g = 1.20, 95% CI 0.32 to 2.09, p = 0.028) subgroups. The between-technique test was not significant (Qbetween = 0.27, p = 0.873); the data therefore did not identify a superior technique.

The meta-regression slope for duration was β = -0.03 per additional month (95% CI -0.15 to 0.08, p = 0.552). Descriptively, pooled effects were significant for both ≤3 months (k = 8, g = 1.37, 95% CI 0.80 to 1.93) and >3 months (k = 8, g = 1.11, 95% CI 0.74 to 1.48), while the subgroups did not differ (Qbetween = 0.89, p = 0.347).

Parent involvement (Qbetween = 1.12, p = 0.289) and comparator type for autism-related outcomes (Qbetween = 0.94, p = 0.331) were also not significant moderators.

### 3.5 Heterogeneity

Heterogeneity was I² = 59.6% for social communication and I² = 77.0% for autism-related outcomes. Their 95% prediction intervals were -0.35 to 1.63 and 0.14 to 2.31, respectively.

### 3.6 Publication bias and sensitivity analyses

The autism-related synthesis contained 16 independent studies. Egger’s intercept was 1.39 (SE = 2.52; t = 0.55; df = 14; p = 0.591), and the test did not detect funnel-plot asymmetry. Formal asymmetry tests were not performed for syntheses with fewer than 10 studies. In leave-one-out analyses, pooled estimates ranged from g = 1.15 to 1.30 for autism-related outcomes and from g = 0.49 to 0.74 for social communication. Every leave-one-out estimate remained positive.

## 4 Discussion

### 4.1 Primary effect estimates for communication, language, and autism-related measures

Three findings are central. First, social communication/interaction showed a moderate significant pooled effect, and the estimate remained significant in the primary evidence layer. Second, the expanded language synthesis showed a significant small effect; the primary evidence layer produced a smaller estimate whose confidence interval included zero. Third, autism-related scales produced a large pooled standardized effect that remained significant among studies reporting reproducible randomization. Adaptive function and quality-of-life estimates did not reach statistical significance.

These findings partly converge with earlier syntheses. A review found no clear immediate differences in generalized social interaction or verbal and nonverbal communication, but reported more favorable evidence for global improvement, quality of life, and overall autism-related severity (15). Ke et al. (58), Yang et al. (59), and Wu et al. (60) also reported favorable pooled effects for several autism-related or behavioral outcomes. The newly incorporated trials and crossover studies extend this literature with relational communication, group social skills, sign and speech imitation, and developmental speech-language training (30, 35, 38, 41, 42). Differences among reviews reflect outcome definitions, comparator intensity, eligibility criteria, search periods, and inferential methods.

The results define benefit by outcome rather than by a single omnibus claim. Social communication, task-based language, and autism-related scale outcomes showed favorable averages; adaptive function and quality of life did not. A lower autism-related score is not interchangeable with greater participation or quality of life, and a task-proximal speech gain does not by itself establish generalized communication.

### 4.2 Theory-based interpretation of moderators

Moderator findings can be interpreted within a theory of change. One plausible pathway has four stages: musical preference, sensory tolerability, and prior experience determine access; shared timing, musical contingency, child initiation, and therapist responsiveness shape proximal interaction; repetition and parent or teacher use influence consolidation and transfer; and reporter, measurement distance, and goal-outcome alignment determine whether change is detected. Technique labels and calendar duration are distal proxies for this pathway.

#### 4.2.1 Technique and engagement mechanism

Pooled effects were significant within all three technique subgroups, while the between-technique test was not. The categories shared components: active work includes listening, imitation, and therapist-provided structure; receptive programs can require prediction, attention, and social response; and mixed programs span both. More plausible active ingredients include child choice and initiation, timely therapist response, predictable but flexible social contingency, and task demands matched to ability. This interpretation is consistent with research on joint attention, therapist responsiveness, and auditory-motor coupling (12, 20, 22, 35, 38). Future trials should code these microprocesses, therapist qualifications, and fidelity rather than compare broad labels alone.

#### 4.2.2 Intervention duration and dose

Calendar duration did not predict effect size, and the descriptive duration groups did not differ. Calendar time combines different session lengths, weekly frequencies, cumulative sessions, attendance, and fidelity. Change may follow a nonlinear course, with a minimum exposure needed to establish a relationship, rapid early gains, a plateau, or delayed transfer after repeated sessions. Trials should report minutes per session, weekly frequency, cumulative sessions, spacing, attendance, and fidelity, and should use repeated measurements or individual-participant data (15, 16, 19).

#### 4.2.3 Comparator intensity

Comparator intensity defines the estimate. For social communication, the point estimate was 0.74 against usual or enhanced care and 0.55 against an active non-music comparator; the between-group test was not significant. Usual-care comparisons estimate the value of a package that includes therapist attention, structure, expectancy, and music, whereas intensity-matched active controls come closer to estimating the incremental contribution of music (12, 29, 35, 38, 45). These studies are particularly informative: both music and structured speech training improved target-word production, but the music-versus-speech contrasts were small (41, 42). Trials should specify whether the target is effectiveness of the full clinical package or the mechanism attributable specifically to music.

#### 4.2.4 Parent involvement and developmental stage

Parent involvement was not a significant moderator, but only three clearly parent-child studies were available and heterogeneity was substantial. Parents may promote transfer by repeating musical routines, recognizing child signals, and creating opportunities for communication; conversely, unblinded parent reports may be sensitive to expectancy, and home practice may add burden. Family-centered and parent-child studies offer preliminary support for this transfer pathway (22, 23). Future trials should quantify home practice and parent fidelity, caregiver burden and family quality of life, and independently assessed child outcomes. Parent involvement is better conceptualized as a potential transfer mechanism than as a binary treatment label.

Age was not analyzed as a quantitative moderator, but development is unlikely to be irrelevant. More proximal candidate moderators include baseline communication mode, intellectual and adaptive functioning, sensory seeking or avoidance, sensitivity to music reward, prior musical experience, child choice, therapist-child fit, and alignment between goals and measurement. Neurodiversity scholarship places preference and acceptability early in this pathway (2, 3): an intervention is unlikely to help when music is unwanted or the sensory load is excessive. Individual differences in music reward and affective processing also support preference as a candidate effect modifier (9, 11). These hypotheses require prespecification, a limited set of tests, and validation with individual-participant data or stratified randomization.

### 4.3 Limitation and future directions

While this meta-analysis provides evidence supporting the benefits of music therapy for autistic children, several limitations should be considered. First, the methodological heterogeneity across included studies—particularly in support protocols and outcome measurements—may affect the comparability of results. Although we explored key moderators, unmeasured variations in therapeutic techniques and practitioner experience remain a source of variability. Second, the generalizability of findings may be limited by the geographical distribution of existing research, which influences the diversity of cultural and support contexts represented. Third, and most importantly, many of the included studies used outcome measures that were not originally designed to align with music therapy’s actual goals (e.g., autism symptom checklists rather than well-being or quality-of-life measures). As noted by Quintin (11), this misalignment has unfortunately contributed to a poor reputation for music therapy in some research circles. In this review, we therefore retained these measures as a separate domain of autism-related characteristics and support needs rather than interpreting them as direct indicators of well-being or quality of life.

Future research should prioritize: (a) developing standardized reporting guidelines for music therapy support that include clear descriptions of support goals, techniques, and intended outcomes; (b) validating outcome measures that capture well-being, quality of life, social connectedness, and self-determined outcomes from the perspectives of autistic individuals and their families; (c) investigating the active components of music therapy through component-control studies; (d) expanding research into underrepresented populations and cultural contexts; and (e) examining the long-term maintenance of benefits. Finally, future research should actively involve autistic people in the design, conduct, and interpretation of studies to ensure that research priorities and outcome measures reflect what autistic individuals themselves value.

## 5 Conclusions

Music-based support showed significant average effects on social communication/interaction, language, and autism-related characteristics and support needs. The primary evidence layer confirmed the social-communication effect but not the language effect. Adaptive function and quality-of-life estimates did not reach statistical significance, and no technique, duration, parent-involvement, or comparator category showed a significant between-group moderator effect. Clinical value should be judged against the child’s preferences, individualized goals, and acceptability while measuring participation, relationships, quality of life, autonomy, and clinically important support needs.

## References

[1] Kapp SK Gillespie-Lynch K Sherman LE Hutman T . Deficit, difference, or both? Autism and neurodiversity. Dev Psychol. (2013) 49:59–71. doi: 10.1037/a0028353 22545843

[2] Leadbitter K Buckle KL Ellis C Dekker M . Autistic self-advocacy and the neurodiversity movement: Implications for autism early intervention research and practice. Front Psychol. (2021) 12:635690. doi: 10.3389/fpsyg.2021.635690 33912110 PMC8075160

[3] Pellicano E den Houting J . Annual research review: Shifting from "normal science" to neurodiversity in autism science. J Child Psychol Psychiatry. (2022) 63:381–96. doi: 10.1111/jcpp.13534 34730840 PMC9298391

[4] Green J . Debate: Neurodiversity, autism and healthcare. Child Adolesc Ment Health. (2023) 28:438–42. doi: 10.1111/camh.12663 37438108

[5] Bonnel A McAdams S Smith B Berthiaume C Bertone A Ciocca V . Enhanced pure-tone pitch discrimination among persons with autism but not Asperger syndrome. Neuropsychologia. (2010) 48:2465–75. doi: 10.1016/j.neuropsychologia.2010.04.020 20433857

[6] Heaton P Hermelin B Pring L . Autism and pitch processing: A precursor for savant musical ability? Music Percept. (1998) 15:291–305. doi: 10.2307/40285769

[7] Mottron L Peretz I Menard E . Local and global processing of music in high-functioning persons with autism: Beyond central coherence? J Child Psychol Psychiatry. (2000) 41:1057–65. doi: 10.1111/1469-7610.00693 11099122

[8] Stanutz S Wapnick J Burack JA . Pitch discrimination and melodic memory in children with autism spectrum disorders. Autism. (2014) 18:137–47. doi: 10.1177/1362361312462905 23150888

[9] Caria A Venuti P de Falco S . Functional and dysfunctional brain circuits underlying emotional processing of music in autism spectrum disorders. Cereb Cortex. (2011) 21:2838–49. doi: 10.1093/cercor/bhr084 21527791

[10] Paul A Sharda M Menon S Arora I Kansal N Arora K . The effect of sung speech on socio-communicative responsiveness in children with autism spectrum disorders. Front Hum Neurosci. (2015) 9:555. doi: 10.3389/fnhum.2015.00555 26578923 PMC4624858

[11] Quintin E-M . Music-evoked reward and emotion: Relative strengths and response to intervention of people with ASD. Front Neural Circuits. (2019) 13:49. doi: 10.3389/fncir.2019.00049 31619969 PMC6759939

[12] Sharda M Tuerk C Chowdhury R Jamey K Foster N Custo-Blanch M . Music improves social communication and auditory-motor connectivity in children with autism. Transl Psychiatry. (2018) 8:231. doi: 10.1038/s41398-018-0287-3 30352997 PMC6199253

[13] Bottema-Beutel K Crowley S Sandbank M Woynaroski TG . Adverse event reporting in intervention research for young autistic children. Autism. (2021) 25:322–35. doi: 10.1177/1362361320965331 33076682 PMC7870528

[14] Sandbank M Bottema-Beutel K Crowley LaPoint S Feldman JI Barrett DJ Caldwell N . Autism intervention meta-analysis of early childhood studies (Project AIM): Updated systematic review and secondary analysis. BMJ. (2023) 383:e076733. doi: 10.1136/bmj-2023-076733 37963634 PMC10644209

[15] Geretsegger M Fusar-Poli L Elefant C Mössler KA Vitale G Gold C . Music therapy for autistic people. Cochrane Database Systematic Rev. (2022) 5:CD004381. doi: 10.1002/14651858.CD004381.pub4 35532041 PMC9082683

[16] Whipple J Schwartzberg ET . Approaches, goals, and scientific integrity in music therapy research with individuals on the autism spectrum: An integrative review. J Music Ther. (2024) 61:386–430. doi: 10.1093/jmt/thae014 39021148

[17] Fuller EA Kaiser AP . The effects of early intervention on social communication outcomes for children with autism spectrum disorder: A meta-analysis. J Autism Dev Disord. (2020) 50:1683–700. doi: 10.1007/s10803-019-03927-z 30805766 PMC7350882

[18] Bruscia KE . Defining music therapy (3rd ed.). Dallas, TX: Barcelona Publishers (2014).

[19] Mayer-Benarous H Benarous X Vonthron F Cohen D . Music therapy for children with autistic spectrum disorder and/or other neurodevelopmental disorders: A systematic review. Front Psychiatry. (2021) 12:643234. doi: 10.3389/fpsyt.2021.643234 33897497 PMC8062803

[20] Kim J Wigram T Gold C . The effects of improvisational music therapy on joint attention behaviors in autistic children: A randomized controlled study. J Autism Dev Disord. (2008) 38:1758–66. doi: 10.1007/s10803-008-0566-6 18592368

[21] LaGasse AB . Social outcomes in children with autism spectrum disorder: A review of music therapy outcomes. Patient Related Outcome Measures. (2017) 8:23–32. doi: 10.2147/PROM.S106267 28260959 PMC5325134

[22] Thompson GA McFerran KS Gold C . Family-centred music therapy to promote social engagement in young children with severe autism spectrum disorder: A randomized controlled study. Child: Care Health Dev. (2014) 40:840–52. doi: 10.1111/cch.12121 24261547

[23] He YS Liu GH Zhang YH Xie NM Lin JL Hu RF . Prospective randomized controlled study on the effects of parent-child cooperative music therapy on children with autism spectrum disorder and their mothers. Chin J Contemp Pediatr. (2022) 24:472–81. doi: 10.7499/j.issn.1008-8830.2201105 PMC9154363 35644186

[24] Page MJ McKenzie JE Bossuyt PM Boutron I Hoffmann TC Mulrow CD . The PRISMA 2020 statement: An updated guideline for reporting systematic reviews. BMJ. (2021) 372:n71. doi: 10.1136/bmj.n71 33782057 PMC8005924

[25] Higgins JPT Thomas J Chandler J Cumpston M Li T Page MJ ., editors. Cochrane Handbook for Systematic Reviews of Interventions version 6.4 (updated August 2023). Cochrane (2023). Available online at: https://training.cochrane.org/handbook.

[26] Borenstein M Hedges LV Higgins JPT Rothstein HR . Introduction to Meta-Analysis. Chichester, UK: Wiley (2009).

[27] Egger M Davey Smith G Schneider M Minder C . Bias in meta-analysis detected by a simple, graphical test. BMJ. (1997) 315:629–34. doi: 10.1136/bmj.315.7109.629 9310563 PMC2127453

[28] Arezina CH . The effect of interactive music therapy on joint attention skills in preschool children with autism spectrum disorder. Lawrence, KS: University of Kansas (2011).

[29] Bieleninik Ł Geretsegger M Mössler K Assmus J Thompson G Gattino G . Effects of improvisational music therapy vs. enhanced standard care on symptom severity among children with autism spectrum disorder: The TIME-A randomized clinical trial. JAMA. (2017) 318:525–35. doi: 10.1001/jama.2017.9478 28787504 PMC5817481

[30] Buday EM . The effects of signed and spoken words taught with music on sign and speech imitation by children with autism. J Music Ther. (1995) 32:189–202. doi: 10.1093/jmt/32.3.189

[31] Chen L Tao HM Li H Shao Z Yao L Liao LJ . Empirical study on the clinical effects of music therapy intervention for children with autism. Chongqing Med. (2010) 39:2481–2482,2485. doi: 10.3969/j.issn.1671-8348.2010.18.042

[32] Chen YY Zeng LY Zhang YW . Effects of visual music training on children with autism spectrum disorder. China Community Med. (2022) 34:58–60.

[33] Dănciulescu T Zaharia A . Piano with a twist: A pilot study exploring the preliminary effects of a piano therapy program for children with autism spectrum disorder. Arts Psychother. (2023) 82:101987. doi: 10.1016/j.aip.2022.10198742574925

[34] Farmer KJ . The effect of music vs. nonmusic paired with gestures on spontaneous verbal and nonverbal communication skills of children with autism between the ages 1–5. Tallahassee, FL: Florida State University (2003).

[35] Gattino GS Riesgo RDS Longo D Leite JCL Faccini LS . Effects of relational music therapy on communication of children with autism: A randomized controlled study. Nordic J Music Ther. (2011) 20:142–54. doi: 10.1080/08098131.2011.56693337339054

[36] Ghasemtabar SN Hosseini M Fayyaz I Arab S Naghashian H Poudineh Z . Music therapy: An effective approach in improving social skills of children with autism. Advanced Biomed Res. (2015) 4:157. doi: 10.4103/2277-9175.161584 26380242 PMC4550953

[37] Huang XL . Empirical study on parent-child group music therapy for social difficulties in children with autism. Tianjin: Tianjin Conservatory of Music (2020).

[38] LaGasse AB . Effects of a music therapy group intervention on enhancing social skills in children with autism. J Music Ther. (2014) 51:250–75. doi: 10.1093/jmt/thu012 25053766

[39] Li RL Yang XY Cao CH . Music therapy combined with language training for language rehabilitation of autistic children. Chin J Hearing Lang Rehabil. (2016) 14:220–3.

[40] Li Q Gao C . Application effects of music therapy in rehabilitation treatment of children with autism. China Community Med. (2018) 30:69–70.

[41] Lim HA . Effect of "Developmental Speech and Language Training Through Music" on speech production in children with autism spectrum disorders. J Music Ther. (2010) 47:2–26. doi: 10.1093/jmt/47.1.2 20635521

[42] Lim HA Draper E . The effects of music therapy incorporated with applied behavior analysis verbal behavior approach for children with autism spectrum disorders. J Music Ther. (2011) 48:532–50. doi: 10.1093/jmt/48.4.532 22506303

[43] Liu C-E Chen P-Y Lai T-J Yang S-M . Effects of group-based music therapy combined with speech therapy on language and social skills in preschool children with autism spectrum disorder: An exploratory randomized controlled trial. Res Dev Disabil. (2026) 175:105345. doi: 10.1016/j.ridd.2026.105345 42468411

[44] Nie MD Li X . Influence of music therapy on rehabilitation training of children with autism. China Continuing Med Educ. (2017) 9:192–3.

[45] Rabeyron T Robledo del Canto J-P Carasco E Bisson V Bodeau N Vrait F-X . A randomized controlled trial of 25 sessions comparing music therapy and music listening for children with autism spectrum disorder. Psychiatry Res. (2020) 293:113377. doi: 10.1016/j.psychres.2020.113377 32798927

[46] Shen S Kong JL Yang J . Effects of guided education combined with parent-child collaborative music therapy on children with autism. China Health Care. (2022) 40:172–5.

[47] Sui GH . A study on the efficacy of music therapy for children with autism. Tianjin, China: Tianjin Medical University (2017).

[48] Wan HX . Efficacy of aripiprazole combined with music therapy for children with ASD and co-occurring emotional and behavioral problems. J Heze Med Coll. (2021) 33:10–2.

[49] Wang XH . Impact of music therapy on rehabilitation training of children with autism and ABC scores. Gen Dental Med. (2018) 5:114–5.

[50] Wang J Zhang XY Zhang JL . The effect of music therapy on children with autism. Kaifeng Univ J. (2009) 28:60–1.

[51] Wen CB . Music therapy combined with rehabilitation training under social-story conditions for school-age children with autism. China Community Health. (2022) 34:84–7.

[52] Williams TI Loucas T Sin J Jeremic M Meyer S Boseley S . Using music to assist language learning in autistic children with minimal verbal language: The MAP feasibility RCT. Autism. (2024) 28:2515–33. doi: 10.1177/13623613241233804 38433533 PMC11504703

[53] Yum YN Poon K Lau WK-W Ho FC Sin KF Chung KM . Music therapy improves engagement and initiation for autistic children with mild intellectual disabilities: A randomized controlled study. Autism Res. (2024) 17:2702–22. doi: 10.1002/aur.3254 39508131

[54] Yurteri N Akdemir M . The effect of music therapy on autistic symptoms and quality of life in children with autism spectrum disorder. Anatolian J Psychiatry. (2019) 20:436–41. doi: 10.5455/apd.1250531622318

[55] Zhou YH . Music therapy combined with language training for language rehabilitation in children with autism. China Sanatorium Med. (2018) 27:1157–9.

[56] Zhou QR Jing YZ Gan XY Gu JH Song M . Rehabilitation effects of music therapy on autism spectrum disorder in children. China Primary Health Care. (2017) 31:68–9. doi: 10.3969/j.issn.1001-568X.2017.05.0030

[57] Zhou Z Zhao X Yang Q Zhou T Feng Y Chen Y . A randomized controlled trial of the efficacy of music therapy on the social skills of children with autism spectrum disorder. Res Dev Disabil. (2025) 158:104942. doi: 10.1016/j.ridd.2025.104942 39938171

[58] Ke X Song W Yang M Li J Liu W . Effectiveness of music therapy in children with autism spectrum disorder: A systematic review and meta-analysis. Front Psychiatry. (2022) 13:905113. doi: 10.3389/fpsyt.2022.905113 36276324 PMC9582596

[59] Yang Y Wang Y Li W Su L . Effect of music therapy on children with autism spectrum disorders in the Chinese population: A systematic review and meta-analysis. Front Psychiatry. (2025) 16:1611182. doi: 10.3389/fpsyt.2025.1611182 40787684 PMC12332751

[60] Wu L Zhang X Liu S Gao X Wang J . Effectiveness of music therapy for children with autism spectrum disorder: Meta-analysis and potential biological mechanisms. Front Psychiatry. (2026) 16:1722874. doi: 10.3389/fpsyt.2025.1722874 41694124 PMC12900671

